# Sulfonylureas or biguanides is associated with a lower risk of rheumatoid arthritis in patients with diabetes: A nationwide cohort study

**DOI:** 10.3389/fmed.2022.934184

**Published:** 2022-07-27

**Authors:** Yu-Jih Su, Jing-Yang Huang, Cong-Qiu Chu, James Cheng-Chung Wei

**Affiliations:** ^1^Department of Internal Medicine, Kaohsiung Chang Gung Memorial Hospital and Chang Gung University College of Medicine, Kaohsiung, Taiwan; ^2^Center for Mitochondrial Research and Medicine, Kaohsiung Chang Gung Memorial Hospital, Kaohsiung, Taiwan; ^3^Institute of Medicine, College of Medicine, Chung Shan Medical University, Taichung, Taiwan; ^4^Department of Medical Research, Chung Shan Medical University Hospital, Taichung, Taiwan; ^5^Division of Arthritis and Rheumatic Diseases, Oregon Health and Science University and VA Portland Health Care System, Portland, OR, United States; ^6^Department of Allergy, Immunology and Rheumatology, Chung Shan Medical University Hospital, Taichung, Taiwan; ^7^Institute of Medicine, Chung Shan Medical University, Taichung, Taiwan; ^8^Graduate Institute of Integrated Medicine, China Medical University, Taichung, Taiwan

**Keywords:** diabetes, rheumatoid arthritis, sulfonylureas, biguanides, incidence rate

## Abstract

**Objective:**

Diabetes mellitus (DM) is associated with immune dysregulation, while sulfonylureas or biguanides have been linked to anti-inflammatory mechanisms. In this study, we aimed to examine the occurrence rate of rheumatoid arthritis (RA) among DM patients and its incidence rate between different treatments.

**Methods:**

This cohort study used the Taiwan National Health Insurance Research Database between 1997 and 2013 to evaluate the primary outcomes of the preventive role of sulfonylureas or biguanides in the development of RA. We used the Chi-square test for categorical variables and Cox proportional hazard regression and log-rank test to explore the time for development of RA in DM patients. Logistic regression was adopted to estimate the odds ratio of RA in different dosages of medication exposure.

**Results:**

Our cohort study included 94,141 DM cases. The risk of RA development of non-sulfonylureas/biguanides users among the DM group in each analysis was set as the reference, and the adjusted hazard ratio of RA in DM patients who were using sulfonylureas or biguanides was 0.73 (95% confidence interval 0.60–0.90). Within 1 year before the index date, compared with no-biguanides users, patients with more than 180 days of prescription of biguanides had a significantly lower RA risk. Similarly, the significantly lower risk of RA was still observed in DM patients who had more than 365 days of prescription of sulfonylurea within 2 or 3 years before the index date of first RA visit (all *p* < 0.05).

**Conclusion:**

Our data suggest that sulfonylureas or biguanides are associated with a lower rate of RA development in patients with DM; the effect of biguanides appeared more rapid than that of sulfonylureas, but the sulfonylureas might have a longer effect on lowering RA development incidence.

## Introduction

Diabetes mellitus (DM) involves risks related to metabolism (1) and immunity (2, 3) and results in targeted organ damage of both microvascular (4) and macrovascular (5) events and elevated risks of infection (6). The abrogation of immune pathway has been found to relieve insulin resistance in DM mice (7).

Furthermore, the optimal treatment of DM has been associated with an improvement of general mortality and comorbidities in several large clinical trials (8). Among all the oral medications of DM, sulfonylureas or biguanides are the most used and recognized as cost-effective medications (9). Besides, from the result in the UK Prospective Diabetes Study, 20-year all-cause mortality, the relative risk was 0.87, while intensive glucose control with sulfonylureas or insulin and biguanides (e.g., metformin) significantly improved health outcomes at the 10-year follow-up in overweight patients (10). The benefit of biguanides has been demonstrated for “any DM -related endpoint” and “all-cause mortality” with newly manifested type 2 DM, and it is recognized as the first-line treatment for DM (11).

The pathogenesis of rheumatoid arthritis (RA) involves genetic background, obesity (12), chronic infection (13), and environmental factors such as oral hygiene (14), smoking (15), and diet (16), which can influence oral (17) and gut microbiota (18) and thus indirectly impact the development of RA. Take obesity as an example, as shown in a US study (12) and other studies (19, 20), it accounts for 4.8 cases per 100,000 (52%) of the increased incidence of female RA patients between 1985 and 2007. As mentioned above, tight control of blood sugar with sulfonylureas or biguanides is beneficial to several DM-related endpoints (11, 21), which confers the similarity in the pathogenesis between the RA and DM (22, 23).

The similarities between RA (24, 25) and DM (26, 27) are profound (22, 23). They have similar diverse immune marker involvement (7, 28) and the profound comorbidity of cardiovascular disease (29). The treatment of DM with sulfonylureas or biguanides can improve mortality in autoimmune diseases (27), and antioxidant medication has the potential to have a protective effect on DM mice (30). To determine whether the sulfonylureas or biguanides delay the development of RA, by using a nationwide database, we conducted a cohort study to examine the impact of sulfonylureas or biguanides on the development of RA among DM patients.

## Materials and methods

### Data source

This study was a cohort study using the National Health Insurance Research Database (NHIRD), in which almost 99% of the overall beneficiaries in Taiwan are enrolled. The database contains all insurance claims data, including outpatient visits, emergency visits, and hospitalization. One million subjects were sampled from the overall beneficiaries, and their data were collected from 1997 to 2013. The sampled database was de-identified, and the study was approved by the Institutional Review Board of Chung Shan Medical University Hospital (CSMUH No: CS2-21176).

### Study design and participants

This is a cohort study and our study population consisted of patients with DM, which was identified using a physician's diagnosis with a disease code (ICD-9 CM code: 250) combined with the prescription of glucose-lowering drugs. Patients with any history of type 1 DM or RA were excluded. We also recorded the date of the first prescription of all oral antihyperglycemic agents, such as sulfonylureas, metformin, α-glucosidase, thiazolidinediones, meglitinides, dipeptidyl peptidase-4 inhibitor (DPP-4 inhibitor), and insulin.

#### Eligible patients with type 2 diabetes

**Figure 1:**
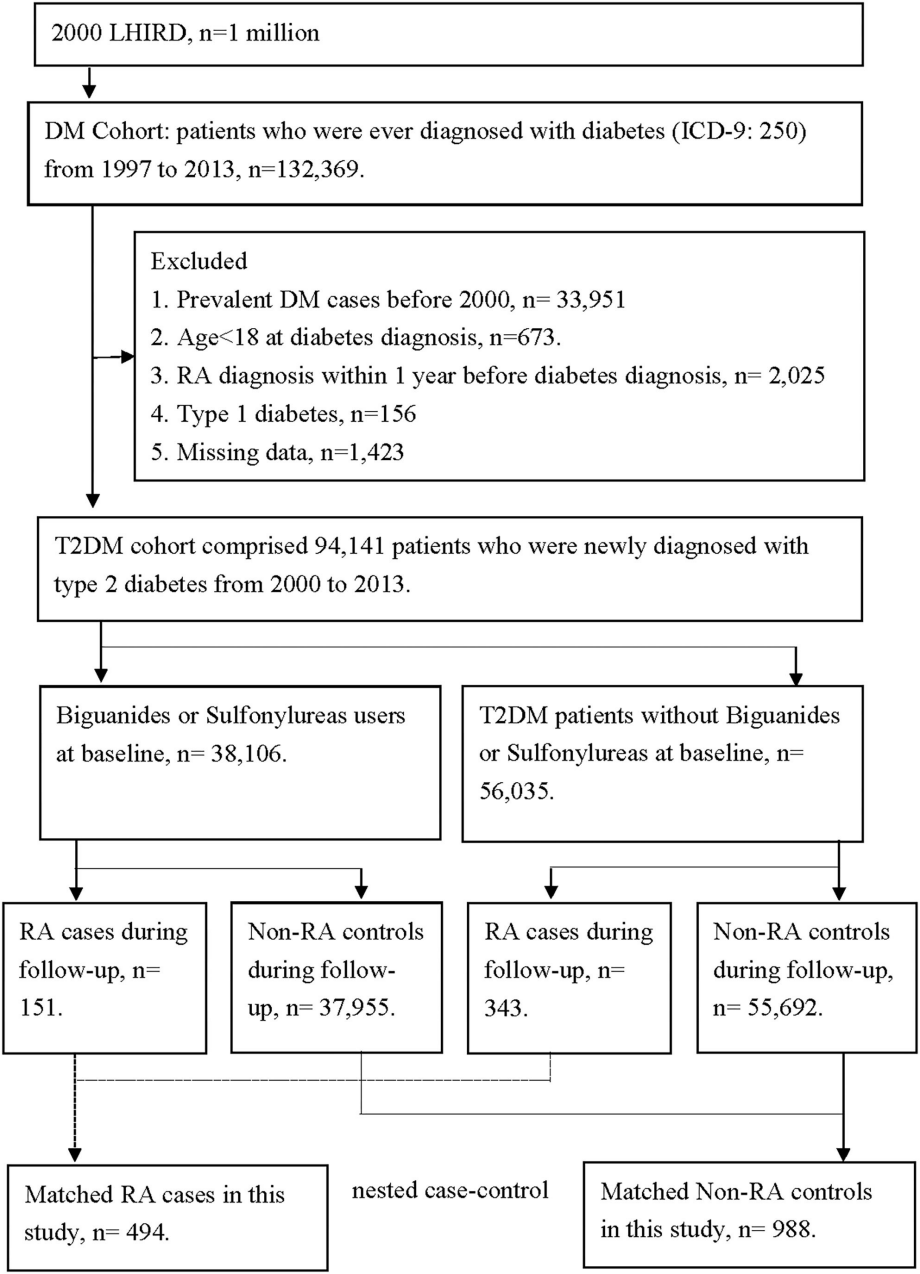
The flowchart of study design. There were 132,369 patients with diabetes in our cohort. We had excluded 33,951 patients for missing data, 673 patients with age <18 years, 2,025 patients with previous RA diagnosis, 2,025 patients with type 1 diabetes, and 1,423 patients with missing age or gender data. So, there were 94,141 diabetes patients enrolled in our cohort. According to the comorbidities, 494 patients were diagnosed with RA, and 93,647 patients were not diagnosed with RA. We had further matched age and gender between the RA patients and non-RA patients. Finally, there were 494 RA patients and 988 non-RA patients in our nested case-control study. The comparisons between the two subgroups in the nested case-control analysis were used to demonstrate the cumulative doses of anti-diabetes treatment and their relation to RA incidence rate.

We enrolled only patients with DM between 1 January 1997, and 31 December 2013, in the cohort with 1 million patients. There were 132,369 patients with DM in our cohort. We had excluded 33,951 patients for missing data, 673 patients with age <18 years, 2,025 patients with previous RA diagnosis, 156 patients with type 1 DM, and 1,423 patients with missing age or gender data, so that there was a total of 94,141 DM patients enrolled in our cohort (flowchart in Figure 1). We also recorded the date of the first prescription of all oral antihyperglycemic agents, such as sulfonylureas, metformin, α-glucosidase, thiazolidinediones, meglitinides, dipeptidyl peptidase 4 (DPP-4) inhibitor, and insulin. We conducted the time-to-event analysis, and all individuals observed in the DM cohort were followed from the first diagnosis of DM until the index date of RA.

#### Identification of primary and secondary outcomes

The goal was to identify the role of sulfonylureas or biguanides in preventing the onset of RA. We selected those RA patients among the DM cohort and the remaining patients were non-RA patients, and the year of diagnosis of RA, birth, and gender were matched with non-RA patients in the subsequent nested case-control study design.

After the enrollment of DM, we identified subsequent occurrence of RA in DM patients by using the corresponding international classification of diseases, ninth revision, clinical modification (ICD-9-CM), and the exact ICD-9 coding will be provided upon requested. Most RA diagnoses were confirmed using a catastrophic illness certification (CIC) according to the NHI program regulations (29). The application of CIC for RA required a strict review process consisting of two rheumatologists, namely, one application rheumatologist and one anonymous senior rheumatologist as an adjudicator. A total of 494 patients had a new onset of RA after DM diagnosis, while 93,647 patients did not. We had further matched age and gender between the RA and non-RA patients. Finally, there were 494 RA patients and 988 non-RA patients in our nested case-control study. For the case of RA, the index date was defined as the first RA visit. For the control of non-RA, the index date was paired with the index date by RA case. The comparisons between the two subgroups in the nested case-control analysis were used to demonstrate the cumulative doses of anti-DM treatment and their relation risk of RA (flowchart in Figure 1). We also recorded exposure to medications, including sulfonylureas and biguanides with at least three prescription medications, namely, biguanides, sulfonylureas, α-glucosidase inhibitors, thiazolidinediones or DPP-4 inhibitors, as well as insulin injections, within 36 months before the index date. The total days of prescription of two major oral antihyperglycemic agents, biguanides and sulfonylureas, were recorded. We confirmed the case of RA development with at least one prescription of oral disease-modification anti-rheumatic drugs. We compared the cumulative probability of RA in each treatment arm of DM and decided whether the duration of each treatment arm affects the RA incidence or not.

The primary outcome of this study was the effect of sulfonylureas or biguanides on RA development (ICD9: 714.0). We conducted time-to-event analysis, and all individuals observed in the DM cohort were followed from the first diagnosis of DM until the index date of RA.

### Identification of covariates

Since the NHIRD does not include laboratory test results (i.e., glycohemoglobin, C-reactive protein), we selected several clinical indicators to represent baseline comorbidities, including hypertension (ICD9: 401–405), hyperlipidemia (ICD9: 272), coronary artery disease (ICD9: 410–414), cerebral vascular accident (ICD9: 430–438), asthma (ICD9: 493), chronic obstructive pulmonary disease (ICD9: 490–492 493–496), chronic kidney disease (ICD9: 585), chronic liver diseases (ICD9: 571, 573), tuberculosis infections (ICD9: 011–018, 137.0), pneumonia (ICD9: 480–486), sepsis (ICD9: 038), herpes zoster (ICD9: 053), and human immunodeficiency virus (HIV) infection (ICD9: 042), which were identified in the 365-day period prior to the index date by using corresponding ICD-9-CM diagnosis codes in the ambulatory record at least twice or in the inpatient record at least once.

### Statistical analysis

To compare the characteristics among study groups, we used the Chi-square test for categorical variables and Cox proportional hazard regression and log-rank test to explore the time to development of RA in DM patients. Logistic regression analysis was adopted to estimate the odds ratio of RA in different dosages of medication exposure. We considered a *p*-value < 0.05 to be statistically significant. Data analysis was conducted using SAS software version 9.4 (SAS Institute, Cary, NC, USA).

## Results

### Eligible patients

We enrolled only patients with DM between January 1, 1997, and December 31, 2013, in the cohort with 1 million patients. There were 132,369 patients with DM in our cohort. We had excluded 33,951 patients for missing data, 673 patients with age <18 years, 2,025 patients with a previous RA diagnosis, 156 patients with type 1 DM and 1,423 patients with missing age or gender data, so that there were 94,141 DM patients enrolled in our cohort. According to the comorbidities, 494 patients were diagnosed with RA, and 93,647 patients did not have RA. We had further matched age and gender between the RA patients and non-RA patients. Finally, there were 494 RA patients and 988 non-RA patients in our nested case-control study. The comparisons between the two subgroups in the nested case-control analysis were used to demonstrate the cumulative doses of DM treatment and their relation to RA incidence rate (flowchart in Figure 1).

### Baseline characteristics for study participants

Table 1 lists the characteristics of sulfonylureas or biguanides users and non-sulfonylureas or biguanides users between DM patients. It also indicates several significant differences of sulfonylureas or biguanides users and non-sulfonylureas of biguanides users between DM patients (all *p* < 0.05), including age, gender, urbanization, income, outpatient visits, length of hospital stay, and some of the comorbidities (Table 1). The differences of the associated comorbidities were listed as follows, including systemic lupus erythematosus, Sjogren syndrome, hypertension, hyperlipidemia, coronary artery disease, cerebral vascular accident, asthma, chronic obstructive pulmonary disease, chronic kidney disease, chronic liver diseases, tuberculosis, pneumonia, sepsis, and herpes zoster (all *p* < 0.001).

**Table 1:** Characteristics at baseline among full diabetes cohort stratified by the use of biguanides or sulfonylureas.

	**T2DM without** **biguanides and** **sulfonylureas** ***n*** = **56,035**	**T2DM with** **biguanides or** **sulfonylureas** ***n*** = **38,106**	***P***
**Age at enrollment**			<0.0001
18–44	11,582 (20.67%)	6,942 (18.22%)	
45–64	27,167 (48.48%)	21,403 (56.17%)	
≥65	17,286 (30.85%)	9,761 (25.62%)	
**Sex**			<0.001
Female	27,673 (49.39%)	16,260 (42.67%)	
Male	28,362 (50.61%)	21,846 (57.33%)	
**Urbanization**			<0.001
Urban	33,394 (59.59%)	21,850 (57.34%)	
Sub-urban	16,157 (28.83%)	11,706 (30.72%)	
Rural	6,484 (11.57%)	4,550 (11.94%)	
**Low income**	430 (0.77%)	210 (0.55%)	<0.001
**Outpatient visits**			
0	566 (1.01%)	762 (2.00%)	
1–27	19,295 (34.43%)	16,148 (42.38%)	
≥28	36,174 (64.56%)	21,196 (55.62%)	
**Length of hospital stay**			<0.001
0	42,918 (76.59%)	32,091 (84.22%)	
1–6	6,040 (10.78%)	2,993 (7.85%)	
≥7	7,077 (12.63%)	3,022 (7.93%)	
**Co-morbidities**			
Ankylosing spondylitis	367 (0.65%)	212 (0.56%)	0.0575
Systemic lupus erythematosus	179 (0.32%)	76 (0.20%)	0.0005
Psoriatic arthritis	31 (0.06%)	20 (0.05%)	0.8543
Sjogren syndrome	616 (1.10%)	246 (0.65%)	<0.0001
Hypertension	28,499 (50.86%)	20,071 (52.67%)	<0.0001
Hyperlipidemia	24,911 (44.46%)	14,741 (38.68%)	<0.0001
Coronary artery disease	13,717 (24.48%)	7,262 (19.06%)	<0.0001
Cerebral vascular accident	8,529 (15.22%)	4,505 (11.82%)	<0.0001
Asthma	7,218 (12.88%)	3,802 (9.98%)	<0.0001
Chronic obstructive pulmonary disease	14,728 (26.28%)	7,121 (18.69%)	<0.0001
Chronic kidney disease	1,803 (3.22%)	518 (1.36%)	<0.0001
Chronic liver diseases	20,765 (37.06%)	10,765 (28.25%)	<0.0001
Tuberculosis	1,507 (2.69%)	667 (1.75%)	<0.0001
Pneumonia	5,608 (10.01%)	2,487 (6.53%)	<0.0001
Sepsis	2,014 (3.59%)	738 (1.94%)	<0.0001
Herpes zoster	2,300 (4.10%)	1,392 (3.65%)	0.0005
**Insulin injection**	483 (0.86%)	916 (2.40%)	<0.0001
**Oral anti-glycemic agents** ^†^			
Biguanides	0 (0.00%)	27,448 (72.03%)	<0.0001
Sulfonylureas	0 (0.00%)	27,636 (72.52%)	<0.0001
Alpha glucosidase inhibitors	624 (1.11%)	1,758 (4.61%)	<0.0001
Thiazolidinediones	254 (0.45%)	1,569 (4.12%)	<0.0001
DPP-4 inhibitors	156 (0.28%)	1,138 (2.99%)	<0.0001

† Usage of anti-glycemic agents is defined as at least 90 days prescription within 1 year from diabetes diagnosis.

Table 1 also shows the differences between the DM patients using sulfonylureas or biguanides and those DM patients who are not using sulfonylureas or biguanides as follows, including the insulin injection, alpha glucosidase inhibitors, thiazolidinediones, or DPP-4 inhibitors (all *p* < 0.001).

### Primary outcomes

The risk of development of RA, stratified by the usage of different anti-DM medications, was summarized in Table 2. The risk of RA development in non-sulfonylureas or biguanides users among the DM group in each analysis was set as a reference, and the adjusted hazard ratio (31) of RA in DM patients who were using sulfonylureas or biguanides was 0.73 [95% confidence interval (29) 0.60–0.90]. The hazard ratio was adjusted for age, sex urbanization, income, length of hospital stays, comorbidities, and use of other anti-glycemic agents.

**Table 2:** Risk of rheumatoid arthritis among diabetes patients.

	**Person-months**	**Rheumatoid** **arthritis events**	Incidence rate^*^ **(95% CI)**	**aHR**^†^ **(95% CI)**
**Any oral anti-glycemic agents at baseline**				
Non-user	4546559	339	0.73(0.65–0.81)	Reference
User	3074179	155	0.50(0.42–0.58)	0.75(0.62–0.91)
**Biguanides or sulfonylureas**				
Non-user	4606873	343	0.73(0.65–0.81)	Reference
User	3013865	151	0.50(0.41–0.58)	0.73(0.60–0.90)
**Biguanides**				
Non-user	5630439	390	0.68(0.61–0.75)	Reference
User	1990299	104	0.51(0.41–0.61)	0.90(0.71–1.15)
**Sulfonylureas**				
Non-user	5235947	374	0.70(0.63–0.77)	Reference
User	2384791	120	0.49(0.40–0.58)	0.82(0.65–1.04)
**Alpha glucosidase inhibitors**				
Non-user	7465536	481	0.64(0.58–0.70)	Reference
User	155202	13	0.82(0.37–1.27)	1.39(0.79–2.43)
**Thiazolidinediones**				
Non-user	7483510	484	0.64(0.58–0.70)	Reference
User	137228	10	0.72(0.26–1.17)	1.44(0.76–2.72)
**DPP-4**				
Non-user	7589832	493	0.64(0.59–0.70)	Reference
User	30906	1	0.32(0.00–0.94)	0.47(0.07–3.38)

* Age, sex-standardized rate, per 10,000 person-months.

† Adjusted for age, sex urbanization, income, length of hospital stay, comorbidities, and use of other anti-glycemic agents.

The incidence of RA was influenced by the DM or by any medication itself (Table 2), which shows that the adjusted hazard ratio of RA in DM patients who were treated with any oral anti-hyperglycemic agents was 0.75 (95% confidence interval 0.62–0.91).

Furthermore, we evaluated the protective effect of each medication used for DM patients in preventing them from developing RA (Table 2). Sulfonylureas or biguanides have no significant effects on reducing the RA risk while either one is used alone, with the adjusted hazard ratio and 95% confidence interval of 0.82 (0.65–1.04) and 0.90 (0.71–1.15), respectively (Table 2).

### Medication usage analysis

Anti-hyperglycemic medication usage at baseline (at least 90 days prescription within 1 year from DM diagnosis) in DM patients is demonstrated in Table 3. Table 3 also shows the percentage of sulfonylureas or biguanides prescribed in this cohort within 3 months of DM diagnosis.

**Table 3:** Usage of anti-glycemic agents at baseline (at least 90 days prescription within 1 year from diagnosis of diabetes) in diabetes patients.

	**DM patients** ***n*** = **94,966**
**Ever use oral anti-glycemic agents**	
Non	55,041 (58.47%)
Yes	39,100 (41.53%)
**Insulin injection**	1,399 (1.49%)
**Ever use oral anti-glycemic agents by Generic name** ^†^	
Biguanides	27,448 (29.16%)
Sulfonylureas	27,636 (29.36%)
Alpha glucosidase inhibitors	2,382 (2.53%)
Thiazolidinediones	1,823 (1.94%)
DPP-4 inhibitors	1,294 (1.37%)

† The sequential prescription of oral anti-glycemic agents was not considered in this analysis. All the oral anti-glycemic agents could be prescribed separately or in combination.

### Subsequent nested case-control analysis

In our subsequent nested case-control analysis, after matching, we obtained 494 RA patients vs. 988 non-RA patients (Table 4). The comparisons between the two subgroups in this nested case-control analysis are demonstrated in Table 4. Demographic data, including DM status, age, gender, and income, did not significantly differ (all *p* > 0.05). Furthermore, Sjogren's syndrome, hypertension, hyperlipidemia, coronary artery disease, cerebral vascular incidents, chronic kidney disease, tuberculosis, sepsis, herpes zoster, and any oral anti-glycemic agents other than sulfonylureas or biguanides (such as α-glucosidase inhibitors, thiazolidinediones, or DPP-4 inhibitors) were all similar between these two subgroups in the nested case-control analysis (all *p* > 0.05) (Table 4).

**Table 4:** Characteristics of diabetes patients with or without rheumatoid arthritis among nested case-control design.

	**Control**, ***n*** = **988**	**Rheumatoid arthritis** **patients**, ***n*** = **494**	***P*** **-value**
**Age at enrollment**			**0.9991**
18–44	114 (11.54%)	57 (11.54%)	
45–64	599 (60.63%)	300 (60.73%)	
≥65	275 (27.83%)	137 (27.73%)	
**Sex**			1.0000
Female	698 (70.65%)	349 (70.65%)	
Male	290 (29.35%)	145 (29.35%)	
**Urbanization**			0.0194
Urban	589 (59.62%)	270 (54.66%)	
Sub-urban	292 (29.55%)	146 (29.55%)	
Rural	107 (10.83%)	78 (15.79%)	
**Low income**	4 (0.40%)	5 (1.01%)	0.1560
**Outpatient visits**			<0.0001
0	6 (0.61%)	2 (0.40%)	
1–27	332 (33.60%)	102 (20.65%)	
≥28	650 (65.79%)	390 (78.95%)	
**Length of hospital stay**			0.0397
0	808 (81.78%)	377 (76.32%)	
1–6	95 (9.62%)	58 (11.74%)	
≥7	85 (8.60%)	59 (11.94%)	
**Co-morbidities at enrollment**			
Ankylosing spondylitis	6 (0.61%)	10 (2.02%)	0.0128
Systemic lupus erythematosus	2 (0.20%)	5 (1.01%)	0.0321
Psoriatic arthritis	1 (0.10%)	4 (0.81%)	0.0266
Sjogren syndrome	9 (0.91%)	9 (1.82%)	0.1312
Hypertension	526 (53.24%)	258 (52.23%)	0.7129
Hyperlipidemia	406 (41.09%)	225 (45.55%)	0.1022
Coronary artery disease	213 (21.56%)	124 (25.10%)	0.1251
Cerebral vascular accident	120 (12.15%)	58 (11.74%)	0.8212
Asthma	106 (10.73%)	79 (15.99%)	0.0039
Chronic obstructive pulmonary disease	212 (21.46%)	147 (29.76%)	0.0004
Chronic kidney disease	19 (1.92%)	13 (2.63%)	0.3764
Chronic liver diseases	317 (32.09%)	203 (41.09%)	0.0006
Tuberculosis	15 (1.52%)	9 (1.82%)	0.6624
Pneumonia	50 (5.06%)	36 (7.29%)	0.0839
Sepsis	15 (1.52%)	6 (1.21%)	0.6411
Herpes zoster	31 (3.14%)	18 (3.64%)	0.6075
**Insulin injection within 1 year before index date** ^**†**^	31 (3.14%)	24 (4.86%)	0.0986
**Any oral anti-glycemic agents within 1 year before index date** ^**†**^	496 (50.20%)	196 (39.68%)	0.0001
Biguanides	376 (38.06%)	158 (31.98%)	0.0217
Sulfonylureas	399 (40.38%)	146 (29.55%)	<0.0001
Alpha glucosidase inhibitors	69 (6.98%)	27 (5.47%)	0.2630
Thiazolidinediones	60 (6.07%)	27 (5.47%)	0.6392
DPP-4 inhibitors	32 (3.24%)	22 (4.45%)	0.2395

† For the case of RA, the index date was defined as the first RA visit. For the control of non-RA, the index date was paired with the index date by RA case.

The variables of urbanization, outpatient visits, length of hospital stay, and some other rheumatoid disease comorbidities, including ankylosing spondylitis, systemic lupus erythematosus, and also asthma, chronic obstructive pulmonary disease, and chronic liver diseases at enrollment were significantly different between the RA and non-RA subgroups in this nested case-control analysis (Table 4, all *p* < 0.05).

### Secondary outcomes

We compared the days of prescription for either biguanides or sulfonylureas in the nested case-control analysis (Table 5). Within 1 year before the index date, compared with no-biguanides users, patients with more than 180 days of a biguanides prescription had a significantly lower RA risk [adjusted odds ratio (32) 0.72; 95% CI 0.53–0.99]. We observed no significant difference of RA risk when comparing the groups classified by days of prescription for biguanides within 2 or 3 years before the index date of first RA visit.

**Table 5:** Nested case-control study design to evaluate the days of prescription for biguanides and sulfonylureas.

	**Control**, ***n*** = **988**	**Rheumatoid** **arthritis patients**, ***n*** = **494**	**aOR (95% C.I.)**
**Duration (Days) of prescription for Biguanides**			
Total amount within 1 year			
Non user	612 (61.9%)	336 (68.0%)	Reference
1–180 days	178 (18.0%)	85 (17.2%)	0.97 (0.71–1.31)
>180 days	198 (20.0%)	73 (14.8%)	0.72 (0.53–0.99)
*p* for trend			0.0556
Total amount within 2 year			
Non user	575 (58.2%)	319 (64.6%)	Reference
1–365 days	240 (24.3%)	110 (22.3%)	0.89 (0.68–1.18)
>365 days	173 (17.5%)	65 (13.2%)	0.75 (0.54–1.05)
*p* for trend			0.0864
Total amount within 3 year			
Non user	549 (55.6%)	303 (61.3%)	Reference
1–365 days	226 (22.9%)	109 (22.1%)	0.95 (0.72–1.25)
>365 days	213 (21.6%)	82 (16.6%)	0.78 (0.58–1.06)
*p* for trend			0.1274
**Duration (Days) of prescription for Sulfonylureas**			
Total amount within 1 year			
Non user	589 (59.6%)	348 (70.5%)	Reference
1–180 days	179 (18.1%)	73 (14.8%)	0.73 (0.53–1.01)
>180 days	220 (22.3%)	73 (14.8%)	0.61 (0.45–0.83)
;p for trend			0.0008
Total amount within 2 year			
Non user	556 (56.3%)	332 (67.2%)	Reference
1–365 days	235 (23.8%)	98 (19.8%)	0.72 (0.54–0.96)
>365 days	197 (19.9%)	64 (13.0%)	0.60 (0.43–0.83)
*p* for trend			0.0007
Total amount within 3 year			
Non user	537 (54.4%)	321 (65.0%)	Reference
1–365 days	218 (22.1%)	93 (18.8%)	0.75 (0.56–1.01)
>365 days	233 (23.6%)	80 (16.2%)	0.62 (0.46–0.84)
*p* for trend			0.0011

aOR, adjusted for age, sex urbanization, income, length of hospital stay, and comorbidities before index date.

On the contrary, the days of prescription for sulfonylureas within 1 year were significantly associated with the risk of RA (aOR 0.61, 95% CI 0.45–0.83), and the *p*-value for trend is statistically significant (*p* = 0.0008). Furthermore, the significantly lower risk of RA was still observed in DM patients who had more than 365 days of a sulfonylurea's prescription within 2 or 3 years before the index date of first RA visit (aOR 0.60, 95% CI 0.43–0.83 within the first 2 years, p for trend is 0.0007; aOR 0.62, 95% CI 0.46–0.84 within the first 3 years, *p* for trend is 0.0011) (Table 5).

## Discussion

**Figure 2:**
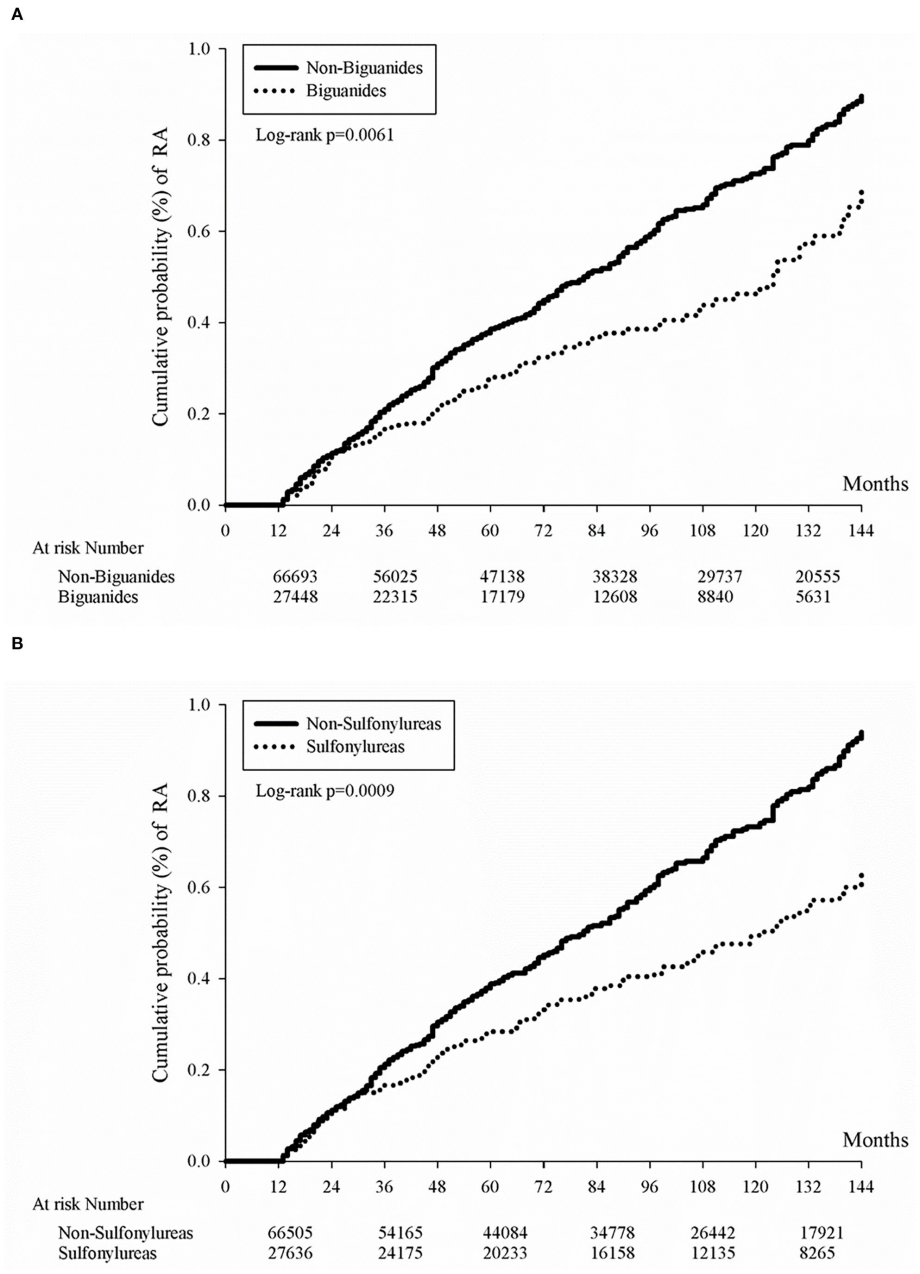
Kaplan-Meier curves of cumulative rheumatoid arthritis incidence proportion in diabetes patients by oral anti-glycemic agent. Kaplan-Meier curves of cumulative rheumatoid arthritis incidence proportion in diabetes patients by the use of either biguanides **(A)** or sulfonylureas **(B)**. Either biguanides users or sulfonylureas users had lower cumulative rheumatoid arthritis incidence rates (p = 0.0061 for biguanides users and p = 0.0009 for sulfonylureas users).

We have previously provided evidence that biguanides were associated with a significantly decreased rate of all-cause mortality and annual admissions compared with those who do not take biguanides among general patients with an autoimmune disease in another study (27). In the current study, we have moved on to determine the effect of sulfonylureas or biguanides on reducing the occurrence of RA disease specifically. In general, the use of sulfonylureas or biguanides may have decreased cumulative probability of RA (Figure 2). Furthermore, in our subsequent nested case-control study, we noticed that the RA patients were less frequently prescribed with sulfonylureas or biguanides (Table 4, *p* = 0.0217, *p* < 0.0001, respectively). In the final analysis, the longer duration of prescription of biguanides in the first year, as well as the longer duration of prescription of sulfonylureas in each year of first 3 years of DM, were associated with reduced odds ratio of RA development (all *p* for trend <0.05, Table 5).

Whether the biguanides prescription reduces the incidence of autoimmune disease is still not evident in our current study. We could only see a transient correlation within the first year of RA from the nested case-control study results. From Table 5, RA patients were prescribed with significantly fewer accumulative days of biguanides when compared with non-RA patients within the first year of RA with an adjusted odds ratio of 0.72 and 95% CI 0.53–0.99. As for sulfonylureas, RA patients were continuously prescribed with less sulfonylurea in the 1-, 2-, and 3-year analysis (Table 5), which may be related to its low priority of treatment recommendation and its risk of hypoglycemia events (33). Although sulfonylureas were less frequently prescribed, we were still able to appreciate its low cumulative incidence rate of RA in Figure 2. Nevertheless, the low incidence rate of RA in DM patients in population and genetic studies had been previously described (34, 35), which might be related to mitochondrial haplotype differences in some places other than most European people (36). As for α-glucosidase inhibitors (37), thiazolidinediones (38), or DPP-4 inhibitors (39), some small cohort studies might show benefits for anti-inflammation effects, but no statistical significance was found in reducing the risk of RA. As another study using the same Taiwan national health insurance database demonstrated higher RA incidence in female DM patients (40), an opposite result was noted in another study (35). Here, we provide another point of view that medication might also affect RA incidence in DM patients.

In our current study, it appears that sulfonylureas could lower the incidence rate of RA (Table 5), and we refer that this effect was mainly based on the diagnosis of RA, which requires elevation of acute phase reactant or evidence of chronic inflammation (41), but early control of DM with sulfonylureas could minimize the level of ESR (42), which in turn delays the development of RA. Besides, the use of different classification criteria of RA, 1987 criteria vs. 2010 criteria (43), depends on the inflammatory markers in diagnosing RA, which might be affected by the influence of sulfonylureas in the 2010 criteria. Nevertheless, the advantage of biguanides is generally accepted (27) because it does not cause hypoglycemia, and it might be a reason that it is preferred as oral anti-diabetic medication to sulfonylureas.

Several overlapping risk factors of DM and RA were also considered with surrogate markers. For example, smoking is a risk factor for RA (44, 45) and DM (46), and we have included the chronic obstructive pulmonary disease, as a surrogate marker, into the confounding factors for analysis (Table 4). Diet, such as western diet (47) or a high lipid diet is a well-known risk factor for DM, could also be a risk factor for RA (48). So, we have included the dyslipidemia and chronic liver disease (e.g., fatty liver) as surrogate markers, into the confounding factors for analysis (Table 4). In general, target organ damage in lung or liver might be a predisposing factor to develop RA among DM patients (Table 4). One previous study demonstrated that the statins showed a time-response effect on RA among DM patients (49), which could be an explanation of dyslipidemia and could be treated with statins (Table 4).

This study has some limitations. First, the exact disease activity of RA or severity of DM in our cohort was unknown, as we could only establish diagnosis through a mixture of different ICD-9-CM codes representing different diseases, which could potentially result in selection bias. We cannot measure smoking, alcoholic drinking, diet habit, exercise habit, sleep quality, medications non-reimbursed by the National Health Insurance, and a lot of causal conditions through this database, and this could be either causative to the development of RA or not. Similarly, each medication being used or not could be confounded by the indication or by the contraindication itself. Second, the enrolled patients were mostly Taiwanese, so the external validity may be questionable, and other ethnic groups would need to verify our results. Third, the role of sulfonylureas or biguanides in RA needs further investigation. The advantage of biguanides is that it has a pleiotropic effect on immune diseases (27) and is safe to be used in other inflammatory diseases (26). But, as shown in Table 5, early and prolonged use of sulfonylureas is associated with a lower RA incidence, which needs further investigation because there are currently few studies supporting this fact. Fourth, despite previous studies showing some benefits of α-glucosidase inhibitors on lowering the risk of RA (50), we cannot confirm this in our current study. Nevertheless, our large-scale study may provide insight regarding prescribing sulfonylureas or biguanides in DM to lower the incidence of RA.

## Conclusion

Use of either sulfonylureas or biguanides is associated with a lower rate of RA development among DM patients. Onset of biguanides effect appears to be more rapid than that of sulfonylureas in lowering RA development. This finding deserves further investigation in populations of other ethnic background. Long-term use of sulfonylureas was found to reduce RA risks, suggesting that sulfonylureas or biguanides lower RA development in this cohort study. This finding deserves further investigation in the future.

## Data availability statement

The original contributions presented in the study are included in the article/supplementary material, further inquiries can be directed to the corresponding author/s.

## Ethics statement

The Institutional Review Board of Chung Shan Medical University Hospital approved this study (CSMUH No: CS2-21176). Informed consent was not required because the datasets were devoid of personally identifiable information.

## Author contributions

C-QC and JW conceived the study. Y-JS and J-YH conducted the literature review, data analysis, and drafted the manuscript. J-YH and JW have full access to all the data in the study, take responsibility for the integrity of the data, and the accuracy of the data analysis. All authors contributed to the acquisition of research data, contributed to revising the article critically for important intellectual content, and approved the final version for publication.

## Funding

This study was supported by research grants from Chung Shan Medical University Hospital grant number CSH-2020-C-007 and Chang Gung Memorial Hospital grant number CRRPG8K0063. The study of C-QC was supported by an Innovative Award from the American College of Rheumatology Research Foundation and by a VA Merit Review grant (I01BX005195).

## Conflict of interest

The authors declare that the research was conducted in the absence of any commercial or financial relationships that could be construed as a potential conflict of interest.

## Publisher's note

All claims expressed in this article are solely those of the authors and do not necessarily represent those of their affiliated organizations, or those of the publisher, the editors and the reviewers. Any product that may be evaluated in this article, or claim that may be made by its manufacturer, is not guaranteed or endorsed by the publisher.
